# Protumor Activities of the Immune Response: Insights in the Mechanisms of Immunological Shift, Oncotraining, and Oncopromotion

**DOI:** 10.1155/2013/835956

**Published:** 2013-03-14

**Authors:** G. K. Chimal-Ramírez, N. A. Espinoza-Sánchez, E. M. Fuentes-Pananá

**Affiliations:** ^1^Unidad de Investigación Médica en Enfermedades Infecciosas y Parasitarias (UIMEIP), Hospital de Pediatría Centro Médico Nacional Siglo XXI, Instituto Mexicano del Seguro Social, Avenida Cuauhtémoc 330, Colonia Doctores, 06725 Delegación Cuauhtémoc, DF, Mexico; ^2^Programa de Doctorado en Ciencias Quimicobiológicas del Instituto Politécnico Nacional (IPN), Mexico; ^3^Programa de Doctorado en Ciencias Biomédicas de la Universidad Autónoma de México (UNAM), Mexico

## Abstract

Experimental and clinical studies indicate that cells of the innate and adaptive immune system have both anti- and pro-tumor activities. This dual role of the immune system has led to a conceptual shift in the role of the immune system's regulation of cancer, in which immune-tumor cell interactions are understood as a dynamic process that comprises at least five phases: immunosurveillance, immunoselection, immunoescape, oncotraining, and oncopromotion. The tumor microenvironment shifts immune cells to perform functions more in tune with the tumor needs (oncotraining); these functions are related to chronic inflammation and tissue remodeling activities. Among them are increased proliferation and survival, increased angiogenesis and vessel permeability, protease secretion, acquisition of migratory mesenchymal characteristics, and self-renewal properties that altogether promote tumor growth and metastasis (oncopromotion). Important populations in all these pro-tumor processes are M2 macrophages, N2 neutrophils, regulatory T cells, and myeloid derived suppressor cells; the main effectors molecules are CSF-1, IL-6, metalloproteases, VEGF, PGE-2, TGF-**β**, and IL-10. Cancer prognosis correlates with densities and concentrations of protumoral populations and molecules, providing ideal targets for the intelligent design of directed preventive or anticancer therapies.

## 1. Introduction

**Figure 1:**
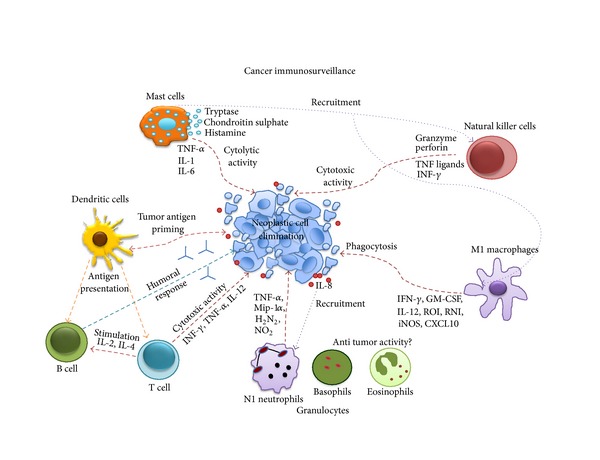
Cancer immunosurveillance activity. Each immune cell fulfills unique and redundant functions to achieve tumor cell elimination. Among the anti-tumor activities found in the tumor microenvironment are cytotoxicity mediated by CD8^+^ T and NK cells, phagocytosis by M1 macrophages, cytolysis induced by mast cells, and humoral responses by B cells. Dendritic cells are primed by tumor antigens, which are then presented to T and B cells for adaptive responses. These activities are coordinated by a variety of molecules secreted by the immune and tumor cells directly to the tumor environment or to circulation where it serves to recruit additional immune populations to the tumor site (see text for details). Red dashed arrows represent direct anti-tumoral effects, blue dashed arrows indirect tumor cell elimination. Dotted arrows represent recruitment of other cell populations.

Somatic cells are in constant risk of cancer transformation and organisms are endowed with surveillance mechanisms carried out by the immune system to control the generation of cancer cells. These mechanisms are (i) controlling infection by oncogenic pathogens, (ii) resolving local inflammation to prevent the establishment of a tumorigenic chronic inflammatory microenvironment, and (iii) eliminating potentially transformed cells. The AIDS pandemic and laboratory recombinant technologies have provided plenty of support to the Burnet and Thomas hypothesis of immunosurveillance (Figure 1) [1, 2]. However, increasing understanding of the relationship between the immune system and cancer points out that there is more than one side to this tale, with more recent evidence supporting a role for the immune system in promoting oncogenesis and tumor growth [3, 4]. This duality displayed by the immune system has led to the concept of an “immunological shift” in cancer, in which immune and transformed cells interact in a dynamic process comprising at least five phases: immunosurveillance, immunoselection, immunoescape, oncotraining, and oncopromotion. The first phase represents a functional immune system engaging in protective functions that successfully eliminates aberrant/malignant cells. In the second phase, an equilibrium is reached between tumor cells and immune cells; this phase depends on the mutational rate of the transforming cell, which creates rapidly proliferating clones, resistant to death and/or self-renewal capacities; thus, the immunosurveillance function is incapable of eliminating all aberrant cells and instead selects clones with increasingly tumorigenic properties. With time, the mutational rate/immune selection process allows cells to develop mechanisms to evade immunosurveillance resulting in an equilibrium shift favoring tumor growth. The immunologically shaped tumor clones take advantage of some immune functions to create a microenvironment in which immune cells are switched from anti- to pro-tumoral activities, in a collective mechanism referred to as oncotraining. Among the immune cell functions hijacked by the tumor stroma is the capacity to stimulate immune regulatory activities, turning off classical phagocytic and cytotoxic immune responses while promoting tissue remodeling functions. Together, these processes result in an oncopromoting phase favoring tumor growth, local invasion, and metastasis.

The term immunoediting has recently been coined to describe the dual role of the immune system in cancer. However, this term mostly reflects the differences between tumors developing in immunocompetent or immunodeficient mice [5], and therefore the role of the immune system in selecting tumor clones with evolutionarily advantages. Immunoediting is divided into three stages, (1) elimination, (2) equilibrium, and (3) escape, mainly reflecting the close relationship between immunogenic properties of the cancer cell and the responses they trigger. The last two stages concern the selection of clones in which the cancer immunogenic determinants have been edited to remain invisible to immunosurveillance mechanisms. However, the immune response does more than selection and can directly participate in every step of the carcinogenic process even directly switching off immunosurveillance. Considering the steady increase in the frequency of cancers observed in recent years, an in-depth understanding of the mechanisms of immunological shift is needed. In this paper, we present evidence regarding the pro-tumoral roles of the immune system and discuss how the immune system can be instructed by the tumor stroma to exhibit cancer-promoting functions.

## 2. Anti-Tumoral Immune Responses

Dendritic cells (DCs), macrophages, and mast cells (MCs) constitutively reside in physiologically normal tissues acting as sentinels that monitor the microenvironment in search of stress signals; when tissue homeostasis is compromised they release cytokines, chemokines, reactive oxygen species (ROS), and bioactive mediators, which among many other functions induce mobilization and infiltration of other leucocytes to the injured site in the process of inflammation. In an inflamed tissue, innate immune cells perform diverse and redundant tasks when activated; for instance, both MCs and granulocytes release their preformed granules to kill or inactivate invasive agents, while macrophages, neutrophils, and DCs carry out phagocytosis. NK cells recognize and kill either virus-infected or malignantly transformed cells. Also, all of these populations act as antigen-presenting cells (APCs), although this is the main function of DCs. Macrophages and DCs, that have engulfed the aggressor, mobilize to lymphoid organs to present antigens to adaptive immune cells and the combined action of innate and adaptive immunity leads to the elimination of stress stimuli and resolution of tissue damage.

MCs are present throughout all tissues in which they are traditionally known to function in the first stages of inflammation and during allergic responses. MCs are activated after ligand binding via the Fc*γ*, complement and/or pathogen-associated molecular patterns (PAMP) receptors, releasing bioactive molecules such as histamine, proteases, lipid mediators, cytokines, and chemokines. These molecules are required for direct pathogen killing, recruitment of immune cells, increased angiogenesis, vascular permeability, and degradation of the injured tissue. To date, the participation of MCs in immunosurveillance is controversial, with some of their bioactive molecules reported to have direct anti-tumor activities; for instance, connective tissue MCs are enriched for granules containing tryptase and chondroitin sulphate that may promote a strong local inflammatory response and inhibit metastasis, respectively [6]. Glycoprotein interactions between tumor cells and the tissues they invade are essential for metastasis. Chondroitin sulphate expressed in the tumor stroma competes with these interactions stopping tumor cells from leaving the primary tumor site [7]. Also, histamine is thought to increase the synthesis of prostacyclin, a potent antimetastatic factor in endothelial cells. MC-released TNF-*α*, IL-1, and IL-6 inhibit tumor growth and angiogenesis in melanoma [8, 9]. MC-derived chemokines recruit phagocytic and cytotoxic cells, and the MC-mediated recruitment and survival of eosinophils had a tumor regression effect in a mouse melanoma model [10]. Interestingly, a direct MC phagocytic activity against tumor cells was observed in invasive ductal breast cancer samples [11]. 

Neutrophils are the most abundant circulating polymorphonuclear (PMN) granulocyte; they search for chemotactic signals to direct them to sites of infection or injury. Although neutrophils' half-lives are only of a few hours, they survive much longer in an inflammatory microenvironment. Like MCs, this lineage protects against invading microorganisms and assists in wound healing through releasing of a wide variety of effector molecules stored in cytoplasmic granules, including proteases such as neutrophil elastase, cathepsin G, proteinase-3, metalloproteases 8 and 9 (MMP-8 and -9), antimicrobial molecules such as defensin peptides and ROS, and a number of cytokines (TNF-*α*, IL-1*β*, IL-8, IL-12, among others) [12, 13]. Whether PMNs are able to effectively target their cytotoxic effects on tumor cells is still controversial. Several reports support a polarization of neutrophils to adopt a highly active anti-tumor profile, in which they produce high amounts of proinflammatory cytokines [14–16]. These anti-tumor “N1” neutrophils synthesize higher levels of TNF-*α*, MIP-1*α*, H_2_N_2_, and NO_2_ and show cytotoxic activity against tumor cells both *in vivo* and *in vitro* [17]. However, Gregory and Houghton argue that the increase in cytotoxic activity does not represent a transcriptional switch but rather shows that neutrophils can be activated to various degrees in response to different stimuli [18].

Basophils and eosinophils represent approximately 4% of blood PMNs and play essential roles in allergic and antiparasitic responses; they are not usually present in tissues but are recruited to inflammatory sites. The main content of basophil granules are histamine, chondroitin sulphate, and proteases, while eosinophil granules contain mostly basic major protein, eosinophil cationic protein, peroxidases, hydrolases, and phospholipases. Although both cell types are recruited to tumor stroma, the functions in this environment have not been elucidated yet. Epidemiologic studies have found an inverse relationship between cancer and both allergic disease and parasitic infections, suggesting a protective function for the granulocytes activated by these stimuli [19]. Similarly, an inverse association has been found between IgE levels, the predominant antibody isotype present in allergic and antiparasite responses, and cancer [20]. High- and low-affinity IgE receptors (Fc*ε*RI and CD23, respectively) are present in several populations of the immune system, suggesting several possibilities for anti-tumorigenic functions. 

Macrophages are the main phagocytic cell lineage of the immune system and are classified according to the type of response in which they participate [21]. *Classically activated* (M1) macrophages are activated in response to a microenvironment enriched with Th1 cytokines (IFN-*γ*, GM-CSF, IL-12, ROI, RNI, iNOS, and CXCL10). In these cells, IFN-*γ* and Toll-like receptor (TLR) ligands activate the NF*κ*B signaling pathway turning on a Th1 transcriptional program characterized by high expression of MHC/HLA, IL-12, TNF-*α*, ROS, and NO. *Alternatively activated* (M2) macrophages are formed in response to Th2 cytokines (IL-4, IL-10, IL-13, M-CSF, CCL2, CCL5, CCL22, and HIF-1*α*) and are characterized by the expression of *JMJD3, arginase-1, YM,* and *FIZZ1* genes and secretion of IL-4, IL-10, and IL-13 upon activation, an expression/secretion profile more in tune with tissue remodeling activities. Macrophages often constitute the most abundant innate immune lineage in the tumor mass and their phagocytic activity remains one of the most important immune anti-tumor functions.

NKs have the ability to recognize and lyse a variety of abnormal cells, including tumor, virus infected, antibody bound, allogeneic, and stressed cells. Recognition of target cells by NKs is achieved through inhibitory or activating receptors expressed on plasma membrane and the lysis of the targets occurs only when activating signals outweigh inhibitory signals. There are three known inhibitory receptor families on NK cells surface that recognize MHC-I/HLA molecules: killer cell immunoglobulin-like receptor (KIR) in humans, Ly49 in mice, and CD94/NKG2A present in both human and mice. In agreement with the hypotheses of “missing-self” and “induced-self”, these receptors are very important to the NK anticancer response, since low amounts or absence of MHC-I/HLA molecules tends to be a characteristic of cells undergoing malignant transformation or viral infection [22, 23]. NK cells have two lytic mechanisms: (i) release of granzyme and perforin and (ii) induction of apoptosis through release of TNF ligands. Additionally, NK cells have a number of other anti-tumor activities: IFN-*γ* release inhibits tumor cell proliferation *in vitro*; release of antiangiogenic factors and DC activation can elicit a T-cell-mediated response [24].

DCs are constitutive residents of skin and mucous membranes where they rapidly respond to microenvironmental signals, turning into mature DCs capable of antigen capture and cross-priming to naïve B and T lymphocytes. DCs collect tumor antigens either from phagocytized tumor cells or through a direct mechanism of capture from living tumor cells; hence they are essential initiators of anti-tumor adaptive immune responses. Tumor-derived antigens presented to B and T lymphocytes trigger high-affinity responses with the capacity to generate immunological memory. Upon activation, CD8 cytotoxic T cells (CTLs) directly eliminate tumor cells while CD4 T helper cells (Th) stimulate B cells supporting both humoral and cytotoxic responses. Antibodies against tumor antigens may directly inhibit tumor cells or mark them as targets of a complement or antibody-mediated cellular activities. Th cells can be classified into four main subtypes: Th1, which mainly secrete IFN-*γ*, TNF-*α*, and IL-12, Th2 secrete IL-4, IL-5, and IL-13, Th17 secrete IL-17 and IL-22, and regulatory T cells (Tregs) that secrete IL-10 and TGF-*β* (transforming growth factor-beta) [25]. Macrophages are also activated by IFN-*γ*; thus Th1 responses are defined by cytotoxic and phagocytic activity. Th1, together with Th17, responses are important to establish an inflammatory microenvironment. A significant increase in neoplasias is observed in patients who are CD8^+^ T cell deficient, supporting the key role of CTLs in tumor surveillance. 

Tregs are perhaps the most important immunomodulatory population. Tregs turn off inflammatory and humoral responses after the trigger signal has been eliminated, thus preventing chronic immune stimulation and autoimmunity [26]. IL-10 and TGF-*β*, the most important Treg-secreted cytokines, have a powerful immunosuppressive activity; these cytokines induce arrest in cell cycle of cytotoxic T cells and block DCs maturation, among many other functions [27]. 

## 3. The Role of Chronic Inflammation in the Pro-Tumoral Immune Response

**Figure 2:**
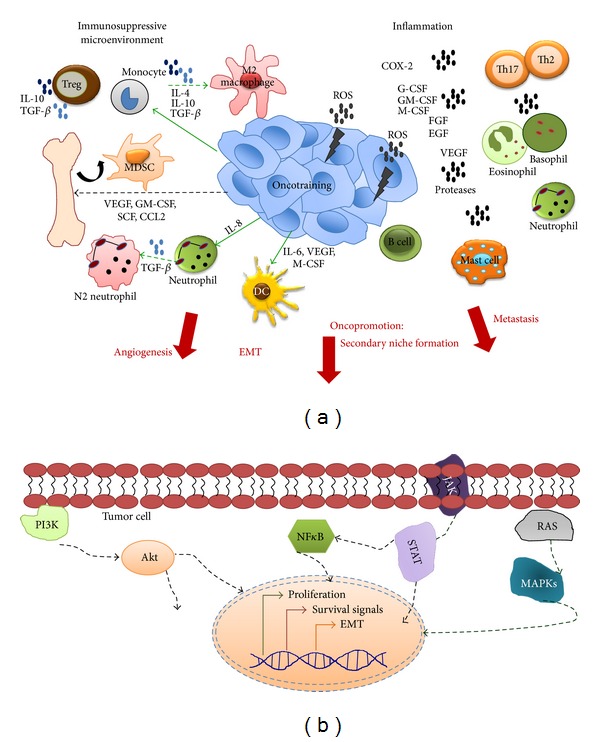
Pro-tumoral activities of the immune system. (a) Soluble factors secreted by tumor and immune cells create a microenvironment in which arriving and local immune cells are (i) inactivated creating immunosuppressive conditions, (ii) maintaining inflammation, and/or (iii) switched from anti- to pro-tumoral activities. Tregs, M2 macrophages, N2 neutrophils, and MDSCs are among the most important immunosuppressive populations and IL-10 and TGF-*β* the main cytokines contributing to this microenvironment. A chronic inflammatory microenvironment contributes to oncogenesis and tumor growth through secretion of mutagenic (e.g., ROS) or inflammatory molecules (e.g., COX-2). Almost all innate immune populations contribute to inflammation plus Th17 and Th2 T and B cells. The anti- to pro-tumoral switch refers to a mechanism in which the tumor microenvironment reprograms or trains the immune cells to perform activities more in tune with the tumor needs (oncopromotion). Polarization to M2 macrophages and N2 neutrophils are perhaps the most studied examples of this process. Among the important molecules for inflammation, oncotraining, and oncopromotion are G-CSF, GM-CSF, and M-CSF (for immune cell recruitment), VEGF (for angiogenesis), proteases (matrix degradation), and TGF-*β* (for EMT). Overall this mechanism contributes to tumor growth, invasion, formation of distant pro-tumoral niches and metastasis (oncopromotion). (b) Intrinsic changes in tumor cells in response to the tumor microenvironment. Signaling from receptors to growth factors, interleukins and other inflammatory molecules activate many pathways. Among the most important are MAPKs and STATs triggering proliferation (e.g., in response to FGF, EGF, HGF and some cytokines), NF*κ*B and PI3K triggering cell survival (e.g., in response to interleukins). Also, one of the most critical mechanisms contributing to tumor malignancy is the transition from epithelial to a more mobile mesenchymal phenotype (EMT) (see text for a detailed explanation).

Tumor initiation and progression are governed by intrinsic mechanism, such as aberrant expression of oncogenes or tumor suppressor genes. Increased evidence points to a critical role for external signals mainly given by immune cells infiltrating chronically inflamed tissue. Injured tissues trigger immune cell recruitment and cytokines, growth factors and other factors secreted by immune cells often promote oncogenic changes, thus creating a positive loop between inflammation and cancer (see Figure 2) [28]. Multiple examples of persistent infections support the crucial role of chronic inflammation in oncogenic processes. In gastric cancer associated with *Helicobacter pylori* infection, oncogenic transformation evolves through progressive stages starting with an inflammatory gastritis, followed by metaplasia, dysplasia, and finally cancer [29]. Similar events have been related to liver cancer associated with hepatitis B and C virus infection. Also, inflammatory autoimmune processes, such as Bowel's disease and prostatitis, trigger the appearance of colorectal and prostate cancer, respectively [3]. An important example of a mutagenic factor frequently enriched in an inflammatory microenviroment is ROS (e.g., oxygen ions and peroxides), which results from the oxidative stress induced by phagocytic cells. ROS are highly reactive molecules that damage DNA augmenting the cell mutation rate, thus favoring the appearance of clones with oncogenic properties or selecting cancer clones with more malignant characteristics [30, 31].

Around 95% of adult cancers are of epithelial origin (carcinomas); epithelium is the outermost layer of organs and functions as a protector barrier against environmental aggressors—chemical (e.g., tobacco components), physical (sun ultraviolet radiation), or biological (infections). An early response of damaged tissues is production of IL-8 by the epithelial cell itself, which together with histamine and TNF-*α*, secreted by resident mast cells and macrophages allows neutrophil extravasation to the injured sites, initiating inflammation [32]. Epithelial cells of the gastric mucosa secrete IL-8 in response to *Helicobacter pylori* infection. Interestingly, this is not just a response to the bacteria gastric colonization but to the presence of the most virulent bacterial factor, the oncoprotein CagA [29].

The leukocyte infiltrate varies in amount, composition, and distribution and each of its components favors cancer at distinct levels. As early as in the 18th century Rudolf Virchow observed that tumors usually have a large leukocyte infiltration. Among malignant characteristics promoted by inflammatory mediators are increased cell proliferation and survival, angiogenesis and vessels permeability, loss of anchorage dependence, and acquisition of a migratory mesenchymal phenotype, the so-called epithelial-mesenchymal transition (EMT), which promotes cancer cell invasiveness and metastasis [33, 34]. Also, tumor cells gain *stemness * characteristics (e.g., self-renewal properties), which often correlates with treatment resistance and high frequency of relapse. Most of these inflammatory pro-tumoral processes are characteristics of tissue repair mechanisms; as it was originally postulated by Virchow: “cancer is a healing process that never stops.” 

Among the inflammatory factors promoting proliferation are TGF-*β*, fibroblast growth factor (FGF), and epithelial growth factor (EGF). TGF-*β* is synthesized by mast cells, macrophages, and lymphocytes as an inactive precursor that is activated by proteases in inflammatory microenvironments. TGF-*β* promotes mesenchymal cell proliferation and facilitates tumor invasion and metastasis of cells that have gone through EMT [35, 36]. The FGF family is a mitogenic trigger of fibroblasts, endothelial and epithelial cells, especially during a healing process. Overexpression of Her2/neu, a member of the EGF receptor family, contributes to over 50% of human breast cancer cases and might also participate in gastric cancer and aggressive forms of uterine cancer [37–39]. Activated macrophages are an important source of both FGF and EGF. IL-6 has also been depicted as a proliferative factor in several types of cancers, and it has been associated with poor prognosis in prostate cancer [40, 41]. Several chemotactic factors such as G-CSF, GM-CSF, and M-CSF can also induce proliferation of innate immune cells at inflammatory sites [42, 43].

Several cytokines, chemokines, growth factors, and TLR ligands trigger signaling cascades that activate the NF*κ*B transcription factor. NF*κ*B is a master regulator of immune responses; it triggers expression of proinflammatory cytokines, adhesion molecules, enzymes, such as cyclooxygenase 2 (COX-2), iNOS, metalloproteases, and angiogenic factors [44]. The NF*κ*B pathway is also an important mediator of cell survival by inducing BCL-2 and Bcl-xL protein expression [45–47]. Nowadays, NF*κ*B and PI3K are recognized as the main mediators of cell survival at inflammatory sites. PI3K is activated by receptors with, or associated with, tyrosine kinase activities (IL-1, -2, -3, -4, -6, and EGF receptors). Activated PI3K phosphorylates and converts phosphatidylinositol (4,5) bisphosphate (PIP2) into phosphatidylinositol (3,4,5) triphosphate (PIP3), which recruits protein kinases such as Akt to membrane-bound signaling complexes. Akt is an important inhibitor of pro-apoptotic proteins, such as BAD and caspase-9, among many other functions [48]. 

Growth factors often target endothelial cells promoting formation of new blood and lymphatic vessels and fulfilling a critical role for tumor maintenance and growth. Vessel formation helps the arrival or exit of O_2_, nutrients, inflammatory factors, and immune cells. Some examples of angiogenic factors are vascular endothelial growth factor (VEGF), EGF, FGF, and hepatocyte growth factor (HGF) [49]. VEGF expression is induced by hypoxic conditions and its principal regulator is the hypoxia inducible factor (HIF-1). HIF-1 is a complex of the *α* and *β* subunits; although both subunits are constitutively expressed, the *α* subunit is constitutively hydroxylated, ubiquitinated, and targeted to the proteasome for degradation in response to O_2_. Therefore, hypoxic conditions suppress HIF-1*α* degradation, promoting stability of the HIF-1 transcriptional complex and resulting in VEGF expression [50, 51]. VEGF promotes the formation of new capillaries by triggering endothelial cell proliferation from preexisting vessels or attracts bone marrow endothelial precursors triggering their differentiation and proliferation [52, 53]. VEGF is also a vasodilator that augments vessel permeability helping the interchange of molecules and cells between the tumor and distant sites. Thus, angiogenesis also facilitates tumor metastasis. VEGF concentration in serum and the number of tumor vessels correlate with cancer prognosis [54]. Currently, VEGF inhibitors are used as therapeutic agents in clinical trials against several neoplasias [3].

Protective barrier functions conferred by epithelial cells depend on cell polarization and formation of protein structures that permit intimate attachments between neighboring cells. These properties are critical for tissue integrity allowing epithelial cells a strong anchorage. One of the most important events in tumor progression is the loss of epithelial features (e.g., cell junction structures, such as E-cadherin, keratin 18, occludins and claudins), and acquisition of more mobile mesenchymal properties (the EMT). It is thought that EMT facilitates detachment of the tumor cell in the primary tumor site, migration and colonization of secondary organs [55, 56]. Inherited mutations in E-cadherin are associated with familial forms of gastric and breast cancer [57, 58]. Moreover, oncogenic pathogens often target this protein [59, 60]: *Helicobacter pylori* oncoprotein CagA interacts with E-cadherin blocking its binding to *β*-catenin; as a result, *β*-catenin accumulates in nucleus where it transactivates gene *p*21^*WAF*1^ important for cell cycle progression, and *CDX1*, which is associated with intestinal metaplasia [61, 62]. CagA-induced E-cadherin loss also correlates with gain of the mesenchymal markers vimentin and fibronectin [63]. Adenovirus 5 destroys cell junctions through interactions with CAR receptor to promote viral exit [64]. Similar mechanisms have been proposed for HPV16 and HBV supporting the importance of E-cadherin loss and EMT [65, 66]. Some inflammatory triggers of EMT are TNF-*α*, TGF-*β*, IL-6, FGF, and EGF [36, 67, 68]. 

Epithelial cells are attached to a basement membrane and extracellular matrix (ECM) of connective tissue, and degradation of both by proteolytic cascades is a key mechanism for tumor cell invasion of surrounding tissues and eventual metastasis. Some of the most important inflammatory proteases are metalloproteases-2 and -9 (MMP-2, MMP-9); serine-, aspartic-, and cystein-proteases (urokinase-like plasminogen activator or uPA, cathepsin D and B, respectively) [69, 70]. MMP-9 is overexpressed in lobulillar breast cancer [71]. Cathepsin B is found at the tumor invasive border supporting its importance for tumor spread [72]. Proteases also activate growth factors and interleukin zymogens. TGF-*β* and plasminogen zymogens are among the most important uPA targets, and high uPA levels are associated with bad prognosis in breast and ovarian cancers [73–75]. In agreement, ECM degradation and cell invasion are reduced by inhibition of cysteine proteases [76, 77]. In murine models of cervix, skin, and pancreatic cancers, VEGF is sequestered by ECM components and angiogenesis only occurs in the presence of metalloproteases [78, 79]. Inhibition of MMP-9 expression by bisphosphonates significantly reduces metastasis to bone in a breast cancer model [80]. Thus, increased expression of proteases and concomitant basement membrane/ECM degradation, EMT, and angiogenesis, all together facilitate tumor metastasis and are poor prognosis markers.

The “seed and soil” hypothesis postulated by Steven Paget in 1889 proposed that tumor cells are systematically released to circulation from the primary site and distributed throughout the body; however, they generate secondary tumors only upon arrival to specific organs. This hypothesis is currently understood as the release of soluble factors by the primary tumor creating the optimal conditions for tumor growth at distant sites. Many functions are fulfilled for those *fertilizing* factors, for example, recruitment of innate immune cells to secondary sites. In a mouse model of breast cancer, RANKL is secreted to circulation by regulatory T cells, promoting migration of RANK-expressing tumor cells to the bone [81]. Also, VEGFR-1^+^ and VLA-4^+^ hematopoietic progenitors migrate from bone marrow to specific tissues prone to secondary growth [82, 83]. In summary, the arrival of soluble factors and different cell populations helps to create a permissive microenvironment for tumor cell colonization, the premetastatic niche, where a secondary tumor will be formed upon arrival of circulating cancer cells.

Proof of the immune cells pro-tumoral role is the positive correlation between their densities at tumor sites and disease prognosis, for example, macrophage and MC numbers correlate with tumor vascularization [84, 85]. In a murine model, attenuation of innate immune cell infiltration prevented the switch from a premalignant to a malignant lesion [86]; also, mice lacking MCs exhibit decreased tumor growth [87]. A similar effect is achieved by blocking macrophage chemotaxis to the tumor with an M-CSF receptor inhibitor [88]. CSF1-deficient mice also show a decreased breast tumor growth and a reduced metastasis to lungs [89]. In humans, CSF1 levels have been shown to correlate with macrophage, tumor density, and poor prognosis [90]. Taken together, all these data argue for an important pro-tumoral role of inflammatory cells infiltrating the tumor stroma. In agreement, COX-2 is an inflammatory protein that is upregulated in several cancers in which it has been associated with bad prognosis [91, 92]. Individuals with excessive blood clotting are frequently treated with periodical amounts of COX-2 inhibitors; these individuals have shown lower rates of breast, colon, lung, and prostate cancer [93, 94]. 

In summary, the immune system is of great help to control cancer through immunosurveillance mechanisms, but it can also trigger cancer promoting mechanisms, and the fine line between both activities is not well defined. In a recent study in mice, injection of flagellin, a TLR5 and NAIP5 ligand, was sufficient to clear tumor cells in a macrophage- and CD8^+^ T cell-dependent manner [95]. It is possible that chronic—rather than acute—inflammation, as it occurs in response to persistent infections, is more in tune with cancer promotion. Therefore, inflammation is recognized as the seventh hallmark of cancer [30].

## 4. Cancer Immunological Shift

### 4.1. Macrophage Oncotraining

There is increasing evidence supporting that the tumor environment instructs cells of the innate and adaptive immune system to shift from anti- to pro-tumoral activities. In this oncotraining process, immune cells entering the tumor stroma lose important anti-tumor activities (e.g., cytotoxicity and phagocytosis), while other immune processes are promoted (e.g. tissue repair activities such as increased cell proliferation and survival, angiogenesis, and EMT) (Figure 2). Tumor-associated macrophages (TAMs) can be phenotypically and functionally divided into two subtypes: M1 or *classically activated* (by Th1 cytokines) and M2 or *alternatively activated* (by Th2 cytokines) also referred to as *killer* or *healer*, respectively [3]. M1 TAMs have immunostimulatory activities; they are accomplished antigen-presenting cells and secrete Th1 cytokines promoting T cell cytotoxic functions, while M2 TAMs are immune suppressors that in homeostatic conditions participate in tissue maintenance and regeneration in case of damage. M2 TAMs also produce CCL22 that attracts Treg cells, which help to maintain an immunosuppressive environment. A shift from M1 to M2 activities normally occurs in an infection episode upon pathogen eradication, and it is thought that in tumoral microenvironments M1 macrophages are often redirected towards M2 functions. 

M2 TAMs promote angiogenesis (by secreting VEGF, angiopoietins 1 and 2, GM-CSF, and EGF), invasion (by secreting proteases MMP-1, -2, -9, cathepsin B and D), and chronic inflammation (by secreting COX-2) [96]. They also protect tumor cells from chemotherapy-induced apoptosis [97]. High densities of M2 TAMs significantly correlate with cancers of poor prognosis; in histological sections of invasive tumors M2 TAMs are preferentially located in areas of basement membrane degradation and increased protease secretion [98–101]. In agreement, knockout mice for the primary tumor macrophage chemoattractant, CSF-1, have a slow tumor growth, low progression to invasive stages, and reduced metastasis [89]. CSF-1 levels are also associated with poor prognosis in patients with breast, ovarian, endometrial, prostate, liver, and colon cancers, and also in several hematological malignancies [97, 102–104].

M2 macrophages also inhibit effector activities of several immune populations, such as M1 macrophages, NK, and cytotoxic T cells, by suppressing the expression of IFN-*γ* and IL-12. IL-10 and TGF-*β* are the main immunomodulatory cytokines and are important effector molecules of M2 macrophages [105]. M2s are normally activated by the resident gut microflora preventing autoimmune colitis [106, 107]. Resident flora-activated M2s secrete TGF-*β* promoting differentiation of T cells into Tregs and triggering apoptosis of activated T cells [108, 109]. Prostaglandin-2 (PGE-2) is secreted by M2 TAMs activated by COX-2 in an inflammatory microenvironment, and PGE-2 also controls differentiation and activation of Tregs [110].

It is documented that TAMs exhibit a high level of plasticity and can shift from M1 to M2 and vice versa, according to the environmental conditions [105]. Condeelis and Pollard showed that there is direct communication between macrophages and tumor cells through receptor-ligand interactions, mainly EGFR-EGF and CXCR4-CSF-1 [111]. Taking all these data together support a model in which macrophages arrive to the tumor site, in which their differentiation into M2 pro-tumor cells is promoted. In this scenario, the cells in the tumor stroma help to create an environment in which immune cells are *reprogrammed* and shifted to functions more in tune with the tumor needs. The consequences of this pro-tumoral shift are immune cells triggering the tumor malignant characteristics, such as increased growth, invasiveness, and metastasis (immune-cell-induced oncopromotion) (see Figure 2). Hence, several authors have proposed to direct anti-tumor therapies against immune modulators of oncotraining and oncopromotion. In this example, drugs directed against M2 macrophages should include inhibiting CSF-1, TGF-*β*, or PGE-2 [112].

### 4.2. Oncotraining of Other Innate Immune Populations

Neutrophils are attracted to the inflammatory site by IL-8 released from the epithelial cell itself in response to oncogenic stress, for instance *Helicobacter pylori* oncoprotein CagA translocation or expression of an oncogenic RAS mutant [113]. Similar to macrophages, neutrophils are polarized in the tumor microenvironment from an anti-“N1” to a pro-tumoral “N2” phenotype, and this shift is mainly regulated by TGF-*β*. Neutrophil depletion with antibodies in experimental models reduced angiogenesis, tumor growth, and metastasis [114]. Accordingly, high densities of neutrophils in renal cell and bronchioalveolar carcinomas are associated with bad prognosis [115, 116]. Interestingly, IL-8 levels also correlate with neutrophil density and reduced survival [113].

Although neutrophils secrete ROS and matrix degrading proteases, it is not clear how they promote tumor growth. Granulocytes are released from bone marrow as mature cells, but in inflammatory processes their myelocytes and promyelocytes precursors also exit to peripheral circulation. Kowanetz and colleagues showed that lung cancer cells secrete G-CSF and mobilize immature cells to premetastatic niches [117]. In these niches, granulocytes secrete prokinectin 2 to attract tumor cells and MMP-9 and induce neighbor cells to secrete VEGF to facilitate the arrival of tumor cells [43]. Granulocytes promote tumor cell proliferation in response to growth factor PDGF [118]. In models of lung adenocarcinoma and mesothelioma, TGF-*β* favors neutrophil N2 accumulation, induces *arginase-1* expression, and inhibits TNF-*α*, CCL3, and ICAM-1 production. N2 neutrophil elimination allows an increase in the activity of cytotoxic T lymphocytes [17]. 

Many tumors are surrounded by mast cells brought to the tumor by SCF (stem cell factor) and other inflammatory chemoattractants, and MCs secrete inflammatory cytokines that in some cases favor tumor growth, angiogenesis, and metastasis [119]. MCs participate in tissue remodeling: activating NF*κ*B through inflammatory mediators, which increases cell survival, and also through suppression of T and NK cell cytotoxic activities [120]. In experimental models, MCs play a decisive role triggering the angiogenic switch preceding malignant transformation, and MC-induced angiogenesis also favors tumor progression in human cancers [121]. Tumor progression stops or is reduced in the absence of MCs [122]. Thus, mast cells and N2 neutrophils have also been proposed as a directed target for cancer therapy [123, 124]. 

The tumor stroma can also suppress immune effector functions without promoting pro-tumoral activities. Different studies support that hypoxia, accumulation of extracellular adenosine and lactate, VEGF, M-CSF, and IL-6, all together, cooperate to inhibit DCs antigen-presenting activity [125–127]. DCs isolated from early stages of mouse ovarian cancer are immunocompetent, but in advanced tumors they fail to activate T cells, correlating with high levels of expression of PDL1, HIF-1*α*, and A2B adenosine receptor [128]. DCs and M2 TAMs have elevated levels of arginase activity [122]. TGF-*β*-enriched microenvironments are also critical for DCs immunosuppression. DCs with high expression of IDO (indoleamine 2,3-dioxygenase) favor the recruitment of T cells; however, the local TGF-*β* induces their differentiation into Tregs, generating an immunosuppressive microenvironment that favors tumor progression [43, 129]. According to these data, depletion of DCs at early time points of tumorigenesis favors tumor progression, but it has a therapeutic effect in advanced tumors.

### 4.3. Oncotraining of Adaptive Immune Cells

70% of solid tumors contain high densities of B lymphocytes, and in preneoplastic lesions (e.g., hyperplasia) they tend to be the most abundant population [130]. The presence of lymphoid follicles in some tumors indicates that the tumor stroma is a center of B cell activation. Furthermore, a positive correlation has been found between the number of B cells secreting IgG or IgM and poor prognosis [131, 132]. To date, it is not clear whether this is a bonafide pro-tumoral mechanism or it is just an indirect effect of the inflammatory microenvironment [133].

A large percentage of solid tumors also have high densities of T cells correlating with either good or bad prognosis [134]. It is proposed that the ratio of CD4^+^ to CD8^+^ T cells is a reliable indicator of prognosis: CD4/CD8 values >1 correlate with poor prognosis and ≤1 with a better prognosis [135]. IFN-*γ* secreted by CD8^+^ T cells helps M1 macrophage polarization and also triggers cytotoxic (by the CD8^+^ T cell itself) and phagocytic (by the M1 macrophage) activities [4]. Th1 CD4^+^ T cells secrete Th1 cytokines enhancing cytotoxic/phagocytic activities, while Th2 CD4^+^ T cells secrete pro-inflammatory cytokines (IL-4, IL-5, IL-6, and IL-13), which inhibit Th1 responses and along with Th17 CD4^+^ T cells promote inflammatory conditions [33]. Treg cells negatively regulate all types of immune responses, innate and adaptive, Th1, Th2, or Th17 [136]. Therefore, CD8 or CD4 Th1 cells favor anti-tumor activities, while Th2, Th17, and Treg populations favor pro-tumor and/or immunosuppressive conditions.

Tregs are characterized by the expression of CD4, CD25, and FOXP3 and two populations have been reported: (i) natural Tregs that mature in thymus and (ii) inducible Tregs, whose differentiation into Tregs is promoted in TGF-*β*-enriched inflammatory environments [137]. The importance of Tregs as pro-tumoral enhancers is based on *in vivo* depletion experiments using anti-CD25 antibodies, in which tumor regression was observed [138]. Tregs exert their immunomodulatory role by secreting TGF-*β* and IL-10 immunosuppressive cytokines, and through cell-cell interactions, mainly via CTLA4. IL-10 and TGF-*β* arrest cell cycle of cytotoxic T cells and block DCs maturation [27]. There is accumulative data that IL-10 is an inhibitor of the JAK/ STAT and NF*κ*B signaling pathways and thereby of expression of IFN-*γ*, IL-2, IL-3, TNF-*α*, and GM-CSF immunostimulatory cytokines [139, 140]. CTLA4 is a T cell inhibitor. T cell activation occurs upon interaction of the TCR and antigenic peptides presented by MHC/HLA molecules in APCs plus CD28 and CD80 or CD86 costimulatory interactions. CTLA4 is also a ligand of CD80 and CD86, thus it competes CD28-CD80/86 interactions inhibiting T cell activation [136]. The CTL4 antagonist, ipilimumab, is currently being used in cancer therapy [141, 142].

### 4.4. Myeloid-Derived Suppressor Cells Oncopromotion Mechanisms

Myeloid-derived suppressor cells (MDSC) are a heterogeneous population of macrophages, granulocytes, DCs, and other early myeloid precursors, which are powerful suppressors of immune cells. Precursor cells with the same phenotype as MDSCs are continuously generated in bone marrow of healthy individuals, in which they differentiate into mature myeloid cells without immunosuppressive activity [143]. Large numbers of CD34^+^ myeloid precursors are present in peripheral blood of patients with head and neck carcinoma and in murine models of lung cancer [144–146]. MDSCs have the ability to suppress innate and T-cell-adaptive immune responses and also to promote tumor angiogenesis, invasion, and metastasis. Two main MDSC populations have been characterized in mice: monocytic MDSCs (CD11b^+^ Gr-1^lo^) and polymorphonuclear MDSCs (CD11b^+^ Gr-1^hi^), also known as granulocytic MDSCs. In cancer patients MDSC are typically Lin^neg^ CD11b^+^ CD33^+^ CD34^+^ CD14^neg^ HLA-DR^neg^ and can vary in their expression of CD15 and other markers [147–149]. Manipulating G-CSF concentration in the tumor microenvironment results in increased accumulation of granulocytic MDSC and tumor growth (high G-CSF) or vice versa [150].The presence of these populations in cancer patients and animal models supports the idea that the tumor microenvironment favors their recruitment and their varied phenotype suggests that different tumors are likely to recruit different subtypes of MDSCs.

The activation and expansion of MDSCs is influenced by factors released by tumor and stromal cells. Within the tumor microenvironment, COX-2, prostaglandins, SCF, CCL2, GM-CSF, M-CSF, VEGF, CXCL5, calcium-binding pro-inflammatory proteins S100A8 and S100A9, and TNF, all favor the chemotaxis and expansion of immunosuppressive MDSCs [151]. STAT3 is arguably the master regulator of MDSCs survival and proliferation, probably through augmenting transcription of BCL-xL, cyclin D1, MYC, and survivin. Persistent activation of STAT3 in myeloid progenitors prevents their differentiation into mature cells and together with the induction of proliferation favors their continuous presence in tumor microenvironments [143, 152]. 

MDSCs immunosuppressive function is activated by IFN-*γ*, TLR ligands, IL-13, IL-4, and TGF-*β*, which trigger STAT3, STAT6, STAT1, and NF*κ*B signaling pathways [153, 154]. Once activated, MDSCs inhibit T cell activation through an RNS (reactive-nitrogen-species-) dependent mechanism of nitrating the T cell antigen receptor (TCR), which dissociates CD3-*γ* from the TCR, preventing antigen/MHC peptide recognition by T cells. MDSCs downregulate arginase synthase and upregulate nitric oxide synthase depleting of arginine and cysteine and increasing nitric oxide, which inhibits T cell activation and proliferation [43, 143, 154, 155]. MDSCs also interrupt T cell migration to lymph nodes by releasing ADAM17, which downregulates the homing receptor CD62L (L-selectin) on T cells [156]. They also inhibit migration of effector CD8^+^ T cells to the tumor by peroxynitrite modification of the chemoattractant CCL2 [157]. MDSCs expand natural Treg cells and promote differentiation of inducible Tregs through their production of IL-10 and TGF-*β*, and through CD40-CD40 ligand interactions [158–160]. MDSCs also inhibit NK cell activity through membrane bound TGF-*β*1, resulting in inhibition of IFN-*γ* and NKG2D expression [161]. MDSCs tumor densities inversely correlate with the anti-tumor activity of cytotoxic T and NK cells [162]. Adoptive transfer experiments of MDSCs into tumor-bearing hosts promote their differentiation into M2 TAMs [43, 163]. MDSCs can also differentiate into DCs, but whether these DCs are immunosuppressed is not currently known [164].

In addition to inhibiting anti-tumor immune responses, MDSCs trigger oncopromotion through facilitating angiogenesis, tissue remodeling, and helping to create premetastatic niches [165]. MDSCs infiltrate the primary site of melanoma promoting cancer cell dissemination by inducing EMT [166]. TGF-*β*, EGF, and HGF are required by MDSCs to induce EMT in cancer cells in *in vitro* assays [166]. MDSCs support the growth of tumor-initiating cells and induce resistance to apoptosis of premalignant cells from the intestinal epithelium [167]. In a murine model of pancreatic cancer, *Kras* oncogene expression at the beginning of the transformation program correlates with accumulation of MDSCs, IL-6, and IL-11 expression, STAT3 activation, increased proliferation, and resistance to apoptosis [168]. In agreement with all these data, a direct correlation between tumor size and the density of MDSCs in tumors have also been found [169] and MDSCs high frequencies in blood have shown correlation with poor prognosis in patients with breast and colorectal cancer [170–172]. 

Ostrand-Rosenberg et al. described a communication network between MDSCs, macrophages, and DCs that promotes and maintains an immunosuppressive microenvironment [173]. This network is critical for oncotraining of tumor arriving immune populations, tipping the balance towards tumor promotion. This communication occurs through inflammatory mediators, mainly IL-1*β*, IL-6, IL-10, PGE-2, and TGF-*β*. MDSCs are also expanded during transplantation and their activity could participate in preventing graft rejection as well as graft-versus-host disease [152, 174]. This is interesting because of the high frequency of malignancies arising in transplanted patients. Because MDSCs seem to play a central role in immunoescape, oncotraining, and oncopromotion, different therapeutic strategies are currently being explored directly targeting these cells. 

## 5. Conclusions

Experimental and clinical studies indicate that cells of the innate and adaptive immune system have dual roles in cancer: the traditional immunosurveillance role and a cancer-promoting role. Immune cell metabolites help to create an optimal environment for tumor growth in which increased proliferation and survival of tumor cells is favored. Immune cells also promote angiogenesis and vessel permeability nourishing and oxygenating the tumor; protease secretion triggering ECM degradation, which together with the high tumor vascularization favor the detachment and exit of tumor cells to distant sites. Immune mediators also help to create the premetastatic niche in which secondary tumors will be formed. Specific immune cell populations have also the capacity to downregulate immunosurveillance mechanisms promoting immunosuppressive environments. In agreement, tumor densities of inflammatory cells correlate with diseases prognosis. All together, these observations support that the tumor stroma shifts immune cells to perform functions more in tune with the tumor needs, a process that is better described as oncotraining, while all pro-tumoral activities resulting from oncotraining are better framed as a mechanism of oncopromotion. More research needs to be done to have a clearer picture of the mechanisms of immunoescape, oncotraining, and oncopromotion, but the important populations in these processes, M2 macrophages, N2 neutrophils, Tregs, and MDSCs, and their main modulators, CSF-1, IL-6, metalloproteases, VEGF, PGE-2, TGF-*β* and IL-10, provide ideal targets for the intelligent design of directed preventive or anticancer therapies. 

Several strategies are envisioned, either by harnessing immunosurveillance or antagonizing mechanisms of oncotraining and oncopromotion. Since plasticity is a common feature of immune cells often exploited by tumors to their own benefit, reprogramming of these populations switching back from pro-tumoral or immunosuppressive to immunosurveillance functions can potentially lead to tumor clearance. In that scenario, a chimeric antibody against IL-6 is in phase II trials in ovarian cancer treatment. Oxaliplatin increases the expression of calreticulin on tumor cells, enhancing phagocytosis, antigen presentation, and ultimately their removal. Adjuvant therapy with bacterial immunostimulatory products has also been helpful. In animal models, antagonistic antibodies against IL-4 or agonistic anti-CD40 favor anti-tumor Th1 functions. Also, the T cell stimulant IL-2 is currently used to treat metastatic renal cancer and melanoma. Alternatively, M2 macrophages, N2 neutrophils, Tregs, and MDSCs pro-tumoral populations can be inactivated or targeted for depletion with specific antibodies. Ipilimumab antagonizes the T cell inhibitor CTLA4 and increases survival of patients with melanoma; antagonists of CXCR4-SDF1 interactions or antagonist of other chemotactic factors (CCL2, CXXL12) are showing promising results inhibiting the arrival of helping populations to primary and secondary tumor sites, thus inhibiting tumor growth and metastasis. A SDF-1 peptide analog has been approved by the FDA for treatment of osteosarcoma. The antiangiogenic drug sunitinib and antibody bevacizumab reduce the numbers of MDSCs in tumors and have also shown anti-tumor activity in preclinical studies. Taking together all these observations, the incorporation of a measure of tumor immunological activity, or an *immunoscore*, has been proposed to be added into the traditional classification schemes of tumor prognosis.

In conclusion, because of the multiple genetic and epigenetic changes that lead a cell to undergo oncogenic transformation, the lesser factors involved in tumor-supportive mechanisms could be more effective targets for therapy, among many types of cancer and even for advanced stages. Still, it is highly unlikely to find a sole mechanism to enhance immunosurveillance while antagonizing oncotraining and oncopromotion mechanisms, but perhaps the combined action of targeted therapy against these mechanisms together with more traditional chemotherapy and radiotherapy will result in more efficient and less toxic cancer treatments.

## Abbreviations

### Cell Populations

APCs: Antigen-presenting cells
CTLs: Cytotoxic T cells
DCs: Dendritic cells
MCs: Mast cells
MDSCs: Myeloid-derived suppressor cells
NKs: Natural killer cells
PMN: Polymorphonuclear cells
TAMs: Tumor-associated macrophages
Th: T helper cells
Tregs: Regulatory T cells
EMT: Epithelial-mesenchymal transition.

### Molecules

ADAM: Metalloproteinase disintegrin
Akt: Protein kinase B
BAD: Bcl-2-associated death promoter
BCL-2: B cell lymphoma 2
Bcl-xL: B-cell lymphomaextra large protein
CagA: Cytotoxin-associated gene A
CAR: Coxsackievirus and adenovirus receptor
CCL: Chemokines with two adjacent cysteines
COX-2: Cyclooxygenase 2
CSF1: Colony-stimulating factor 1
CXCL: Chemokines with N-terminal cysteines separated by one amino acid
CXCR: CXCL chemokine receptor
EGF: Epithelial growth factor
EGFR: Epithelial growth factor receptor
ECM: Extracellular matrix
FGF: Fibroblast growth factor
FOXP3: Forkhead box P3
G-CSF: Granulocyte colony-stimulating factor
GM-CSF: Granulocyte-macrophage colony stimulating factor
HBV: Hepatitis B virus
HCV: Hepatitis C virus
HGF: Hepatocyte growth factor
HIF-1*α*: Hypoxia inducible factor alpha
HLA: Human Leukocyte antigen
HPV16: Human papilloma virus 16
ICAM-1: Intercellular adhesion molecule 1
IDO: Indoleamine 2,3-dioxygenase
IFN-*γ*: Interferon gamma
iNOS: Inducible nitric oxide synthase
KIR: Killer cell immunoglobulin-like receptor
M-CSF: Macrophage colony-stimulating factor
MHC: Major histocompatibility complex
MIP-1*α*: Macrophage inflammatory protein 1 alpha
MMP: Matrix metalloprotease
NAIP5: Neuronal apoptosis inhibitory protein
NF*κ*B: Nuclear factor kappa-light-chain enhancer of activated B cells
NKG2D: Natural killer group 2, member D receptor
PAMP: Pathogen-associated molecular patterns
PDGF: Platelet-derived growth factor
PDL1: Programmed cell death 1 ligand 1
PGE2: Prostaglandin 2
PI3K: Phosphatidylinositide 3-kinases
PIP2: Phosphatidylinositol (4,5) bisphosphate
PIP3: Phosphatidylinositol (3,4,5) triphosphate
RAS: Rat sarcoma proteins
RNS: Reactive nitrogen Intermediates
ROS: Reactive oxygen species
RNI: Reactive nitrogen species
TGF-*β*: Transforming growth factor beta
TNF-*α*: Tumor necrosis factor alpha
TLR: Toll-like receptor
uPA: Urokinase-like plasminogen activator
VLA4: Integrin alpha4beta1(very late antigen-4)
VEGF: Vascular endothelial growth factor
VEGFR: Vascular endothelial growth factor receptor
RANKL: Receptor activator of nuclear factor kappa-B ligand
SCF: Stem cell factor
TCR: T Cell antigen receptor.
